# Peculiar characteristics of new-onset Type 1 Diabetes during COVID-19 pandemic

**DOI:** 10.1186/s13052-022-01223-8

**Published:** 2022-02-09

**Authors:** Concetta Mastromauro, Annalisa Blasetti, Marina Primavera, Lucio Ceglie, Angelika Mohn, Francesco Chiarelli, Cosimo Giannini

**Affiliations:** Department of Pediatrics, University of Chieti, Chieti, Italy

**Keywords:** Type 1 Diabetes, COVID-19, Diabetic ketoacidosis, Children

## Abstract

**Background:**

The COVID-19 pandemic period is having a strong impact on the management of diabetes as well as other chronic diseases as shown by the most severe clinical presentation at onset. The aim of this study was to evaluate the severity of diabetic ketoacidosis (DKA) in youth with newly diagnosed type 1 diabetes in “Santissima Annunziata Hospital” (Chieti, Italy) during COVID-19 pandemic in comparison to the five previous years.

**Methods:**

A retrospective population-based incidence study was performed. Data were obtained from hospital records of 172 patients with new onset type 1 diabetes divided into two groups according to the diagnosis: Group I, between January 2015 and February 2020; Group II, between March 2020 and April 2021. Data regarding anthropometric, socio-economic and laboratory test were analyzed. DKA (pH < 7.30) and different severity of the disease (severe pH < 7.10; moderate pH < 7.20, mild pH < 7.30) were evaluated. A Spearman correlation between pH values and the main variables of interest was performed.

**Results:**

DKA frequency was increased by 19 percentage in Group II compared to Group I (55% vs 36%; *P* = 0.03) with a significant increased risk of severe DKA cases compared to the previous five years (severe DKA 22.5% vs. 8.4%, *P* = 0.01). pH values were significantly related with HbA1c, blood glucose and c-peptide values in all groups. In addition, in Group II but not in Group I, pH values correlated with Triglycerides and TG/HDL cholesterol ratio.

**Conclusions:**

During COVID-19 pandemic the risk of more severe clinical presentation of type 1 diabetes at onset is increased. The correlation with lipid profile might suppose an additional effect of lifestyle changes beside the delay in the diagnosis. Modifications of health care system need to be implemented during this peculiar situation in order to avoid such a relevant complication at onset.

## Background

The first reported case of CoronaVirus Disease 2019 (COVID-19) was in Wuhan City, China in December 2019 [1]. The median age of infected patients was approximately 62 years variable according to the country and period of the study. Interestingly, only 1.5% of patients diagnosed with Severe Acute Resporatory Syndrome-CoronaVirus 2 (SARS-CoV-2) are aged 0–18 years [2]. On March 9^th^ a state of epidemic was announced in Italy and the lockdown period started. Afterwards, the World Health Organization declared a pandemic on March 11, 2020. The Italian Government imposed on March 2020 a national lockdown with travel restriction, closing of school, and suspending elective hospital activities. These recommendations have a strong consequence on different aspects of life such as on management of public health service. Therefore, the accesses to emergency room were strongly recommended to be limited to life-threatening conditions [3]. In addition, the health-care resources and workforce were focused on the growing COVID-19 pandemic with limited access to general pediatrician outpatient. All these changes have led to divert attention from other diseases non COVID-19 related, such as paediatric type 1 diabetes causing delayed identification of new onset diabetes with an increased risk of severe diabetic ketoacidosis (DKA) [4].

Type 1 diabetes (T1D) is one of the most common chronic autoimmune disease during childhood accounting for about 5–10% of all diabetes forms [5]. In the last decades the incidence of disease has increasing in many countries with estimated overall annual increase at around 3% with geographic differences [6]. DKA is one of the most important life-threatening complications of diabetes [7] and is generally the consequence of a misdiagnosis [5] which can be strongly modified by appropriate educational campaigns. In fact, Italian studies have shown a reduction in frequency of DKA following educational campaigns directed to targeting schools and primary healthcare physicians [8]. Therefore, all changes related to the current pandemic period could strongly affect the disease having a strong impact on the management of it as well as other chronic diseases. In this study we aimed to evaluate the impact of COVID-19 pandemic on the frequency and severity of DKA in comparison to five previous years.

## Methods

### Participants

We retrospectively analyzed data from all children with newly diagnosed type 1 diabetes, aged 0 to 18 years, who had been admitted to the Department of Pediatrics of “SS. Annunziata Hospital” sited in Chieti, Italy, between 2015 and 2021. Data were collected from medical records of each patient, retrospectively, and included age, sex, weight, height, body mass index (BMI), date of diabetes onset, main medical history, education, and occupation levels of parents, first-degree relatives with T1D, and laboratory analysis. The University Hospital of Chieti is a regional reference centre for pediatric diabetes at onset. Therefore, all newly diagnosed diabetes children, patients admitted to the emergency rooms of peripheral hospitals as well as subjects evaluated by general pediatricians with a suspected diagnosis of diabetes are sent to the hospital for immediate treatment of acute metabolic disarrangement and subsequently for education in the daily management of the disease.

The population study included 172 children that were then divided into two groups: *Group* I, subjects with diabetes onset between January 2015 and February 2020 (prior to the spread of COVID-19 pandemic); *Group II,* subjects with diabetes onset between March 2020 and April 2021 (during the pandemic period). The average age was 9.3 ± 4.3 and 8.4 ± 4.9 years in the Group I and Group II, respectively. Diabetes diagnosis was made according to the American Diabetes Association Criteria based on a random plasma glucose ≥ 200 mg/dL (11.1 mmol/L) in presence of symptoms of hyperglycemia or hyperglycemic crisis [5]. The autoimmune etiology was confirmed by the presence of two or more auto-antibodies. Furthermore, patients with prediabetes, non-type 1 diabetes or admitted for impaired metabolic control in previously diagnosed diabetes or with diabetes diagnosed in another medical center were not eligible. In addition, DKA was defined according to the International Society for Pediatric and Adolescent Diabetes (ISPAD) guidelines as: a venous pH < 7.30 and/or bicarbonate < 15 mmol/L along with ketonemia or ketonuria and hyperglicemia (blood glucose > 11 mmol/L [≈200 mg/dL]) [4]. As well the severity of DKA was defined according to pH values into three classes: severe DKA (pH < 7.10 or bicarbonate < 5 mmol/L), moderate DKA (pH < 7.20 or bicarbonate < 10 mmol/L), mild DKA (pH < 7.30 or bicarbonate < 15 mmol/L) [4]. The paper is exempt from ethical committee approval since i) it was confined to anonymised and unidentifiable data routinely collected at the SS. Annunziata Hospital (Chieti); and ii) the data analysed for the study are part of routine for patients with diabetes onset admitted to our hospital; and iii) the study findings would not affect patient care.

### Data collection

#### Anthropometric measurements

Height was measured to the nearest 0.1 cm with Harpenden stadiometer [9]. Body weight was measured to the nearest 0.1 kg with a calibrated scale. BMI, used as adiposity index, was calculated as the weight in kilograms divided by the square of the height in meters. Height, weight and BMI z-scores (SDS) were calculated based on the age and sex reference values for the Italian population [10].

#### Socioeconomic status

Parents’ education level was classified as low (without a high school diploma) or < 13 schooling years, and high (high school diploma attainment or university studies) or at least 13 schooling years. Parents’ occupation was collected following the classification of the Italian National Institute of Statistics (ISTAT), which is totally cross-linkable with the International Standard Classification of Occupations. They are grouped into two levels: low (unoccupied, unskilled and semi-skilled workers, manual workers and craftsmen), and high (legislators, senior officials and managers, professionals, technicians and associate professionals, sales workers, small business and farm owners, administrators and higher executives).

#### Biochemical analysis

- Routine basal tests
  In our clinical practice in the emergency room, before the first insulin injection, blood samples are obtained from each patient to assess: plasma glucose, electrolytes, renal function, blood gas analysis with measurement of pH, bicarbonate (HCO3-), B.E. (base excess). During COVID-19 pandemic an oropharyngeal and nasopharyngeal swab test to detect SARS-CoV2 infection is performed in all subjects.
- Additional exams

During hospitalization soon after glycemic control has been established, blood samples are collected to asses glucose metabolism: C-peptide, Hemoglobin A1c (HbA1c), and a panel of islet antibodies (autoantibodies to glutamic acid decarboxylase [GAD65], islet cell autoantibodies [ICA], the tyrosine phosphatases [IA-2]) [11]. Celiac disease screening is also performed for the determination of tissue transglutaminase antibodies IgA and IgG (tTGA‐IgA, tTGA-IgG), and Anty-Endomysial antibodies (EMA‐IgA) in the serum. Thyroid function screening including Thyrotropin (TSH), free T4 (fT4), Thyroglobulin antibodies (Tg-Abs), Thyroid peroxidase antibodies (TPO-Abs) is achieved. TPO-Ab levels higher than 10.1 UI/ml and Tg-Ab levels higher than 28.7 UI/ml are considered positive [12, 13]. Finally, the lipid profile measuring total cholesterol, High-Density Lipoprotein (HDL) cholesterol, Low-Density Lipoprotein (LDL) cholesterol and triglycerides is carried out using an enzymatic calorimetric test. TG/HDL Cholesterol ratio was also determined as index of insulin sensitivity [14].

#### Statistical analysis

All data were expressed as means ± SD. Differences in categorical variables between groups were assessed by the χ2 test. Differences in term of anthropometric parameters and laboratory measurements between *Group* I (subjects with diabetes onset between January 2015 and February 2020) and *Group II* (subjects with diabetes onset between March 2020 and April 2021) were analyzed by Mann–Whitney test. Differences in total DKA cases and in the incidence of different DKA classes of severity between the two groups were analyzed. In addition, differences between severe DKA (pH < 7.1) and all other classes of pH values (pH > 7.1) were also evaluated. As well as the two groups were analyzed by comparing the association of severe and moderate DKA in respect to mild and non DKA. In addition, in order to finally explore the relationship between pH values and different variables of interest in the entire study population and in the two groups of subjects (Group I and Group II), a Spearman correlation was performed. At last, a multiple stepwise backword regression analysis was performed in order to confirm the significative correlation between pH values and main clinical and laboratory parameters in the two groups of subjects. *P* values ≤ 0.05 were considered statistically significant. Statistical analysis was performed using SPSS Program (Statistical Package for Social Science), version 17.0 software for Windows (SPSS, Chicago, IL, USA).

## Results

### Baseline anthropometric and socio-economic characteristics of all subjects with new onset type 1 diabetes divided into two main groups.

The anthropometric and socio-economic characteristics of the two study groups are reported in Table 1. The population study was divided into two groups: *Group I* included 132 children (male/female: 81/51) aged of 9.3 ± 4.3 and *Group II* consisted of 40 subjects (male/female: 20/20) aged of 8.4 ± 4.9 years. No differences for age, sex, weight, height were found. Noteworthy, a significant difference in term of weight SDS, height SDS, BMI and BMI-SDS were documented between the two study groups. One of the subjects in Group II had a COVID-19 disease during hospitalization characterized by mild upper respiratory tract infection without the need for oxygen supplementation and the presence of normal cardiac enzymes and ECG trace.

**Table 1:** Baseline anthropometric and socio-economic characteristics of newly diagnosed children with type 1 diabetes before (Group I) and during (Group II) COVID-19 pandemic

	**Group I**	**Group II**	***P***
**Antropometric Parameters**			
Numerosity (n)	132	40	
Gender (M/F)	81/51	20/20	0.20
Age (years)	9.3 ± 4.3	8.4 ± 4.9	0.27
Weight (kg)	36.7 ± 19.3	32.0 ± 23.0	0.06
Weight-SDS	-0.07 ± 1.31	-0.77 ± 1.47	**0.001**
Height (cm)	135.6 ± 26.0	127.4 ± 31.6	0.15
Height-SDS	0.13 ± 1.20	-0.34 ± 1.32	**0.04**
BMI (kg/m^2^)	18.5 ± 4.4	17.5 ± 5.6	**0.01**
BMI SDS	-0.26 ± 2.27	-0.75 ± 1.58	**0.006**
**Socio-economic characteristics**			
Family history of T1D (yes/no)	10/122 (7.6%)	6/34 (15%)	0.15
Mother education (high/low)	55/77	8/32	**0.01**
Father education (high/low)	62/70	17/23	0.61
Father occupation level (high/low)	51/81	13/27	0.48
Mother occupation level (high/low)	51/81	8/32	**0.01**

Data are mean ± *SD*. Significant values *P* ≤ 0.05. *P* expresses Mann–Whitney analysis across the two groups. *SDS*, standard deviation score; *B.E*., Base Excess. HbA1c, Hemoglobin A1c. IA2, Islet Antigen 2. TG, anti-transglutaminase antibodies. *EMA*, anti-Endomysial antibodies. *TSH*, Thyroid-Stimulating Hormone. *fT4*, free-Thyroxine. *TPO*, Thyroid Peroxidase antibodies. *Tg*, Thyroglobulin antibodies

No significant difference regarding family history of diabetes was documented (*P* = 0.15), although a greater percentage of subjects with positive history of diabetes was reported in Group II than in Group I (15% and 7.6%, respectively). A significant difference in terms of mother’s education (*P* = 0.01) and occupation (*P* = 0.01) was highlighted between the two study groups showing the parents of Group I a higher level of education and occupation than those of Group II. No significant difference was documented for father’s education and occupation between the two study groups.

### Baseline clinical and biochemical characteristics of all subjects with new onset type 1 diabetes divided into two main groups.

The clinical and biochemical characteristics of the study population are reported in Table 2**.** The two groups were similar for bicarbonate, base-excess (B.E), Glycemia, HbA1c, C-peptide. Instead, a significant difference between the two groups was reported for pH values with lower pH mean values in Group II compared to Group I (*P* = 0.01). No differences were found regarding islet autoantibodies positivity. TSH values and thyroid autoantibodies were similar between the two groups while free-T4 values was significantly lower in Group II compared to Group I (*P* = 0.001). A significant difference was found for tTG IgA and tTG IgG positivity showing Group II a higher proportion of tTGA IgA and IgG positivity compared to Group I (*P* = 0.02 and *P* = 0.02, respectively). In contrast, EMA positivity was similar between the two groups (*P* = 0.94). Total and LDL cholesterol were similar between the two groups. HDL cholesterol was lower in Group II compared to Group I, although it did not reach a significant value. In contrast, tryglicerides and Tg/HDL cholesterol values were significantly higher in Group II compared to the Group I (*P* = 0.01 and *P* = 0.004, respectively).

**Table 2:** Baseline clinical and laboratory characteristics of newly diagnosed children with type 1 diabetes before (Group I) and during (Group II) COVID-19 pandemic

	**Group I**	**Group II**	***P***	
**Clinical presentation**				
Severe DKA (pH < 7.1, yes%)	11/132 (8.4%)	9/40 (22.5%)	**0.01**	0.04*
Moderate DKA (pH < 7.2, yes%)	18/132 (13.6%)	6/40 (15%)		
Mild DKA (pH < 7.3, yes%)	19/132 (14.4%)	7/40 (17.5%)		
No DKA (pH > 7.3, yes%)	84/132 (63.6%)	18/40 (45%)	**0.03**	
**Blood Gas Analysis**				
pH	7.28 ± 0.12	7.23 ± 0.14	**0.01**	
HCO3^−^ (mmol/L)	16.8 ± 6.3	14.8 ± 7.8	0.15	
B.E	-10.8 ± 10.4	-13.8 ± 9.7	0.13	
**Glycemic Metabolism**				
Glycemia (mg/dL)	395 ± 143	399 ± 130	0.81	
HbA1c (mmol/mol)	103.7 ± 26	107.0 ± 27.6	0.46	
C-peptide (ng/mL)	0.59 ± 0.56	0.50 ± 0.51	0.12	
**T1D Autoimmunity**				
GAD Ab (U/ml)	279 ± 515	464 ± 1105	0.27	
IA2 Ab (UI/mL)	341 ± 867	111 ± 145	0.11	
Insula Ab (positive/negative)	29/103	14/26	0.09	
**Celiac Screening**				
Anti-TG IgA (positive/negative)	5/127	6/34	**0.02**	
Anti-TG IgG (positive/negative)	2/130	4/36	**0.02**	
EMA (positive/negative)	7/125	2/38	0.94	
**Thyroid Function**				
TSH (µIU/mL)	2.47 ± 1.24	2.34 ± 2.02	0.07	
fT4 (ng/dL)	1.24 ± 0.24	1.04 ± 0.13	**0.001**	
TPO Ab (IU/mL)	0/132	1/39	0.07	
Tg Ab (IU/mL)	0/132	1/39	0.07	
**Lipid profile**				
Total cholesterol (mg/dL)	153 ± 33	158 ± 27	0.47	
HDL Cholesterol (mg/dL)	45 ± 13	40 ± 7	0.14	
LDL Cholesterol (mg/dL)	92 ± 31	100 ± 24	0.30	
Tryglicerides (mg/dL)	94 ± 54	116 ± 61	**0.01**	
Tg/HDL ratio	2.04 ± 1.18	3.17 ± 2.24	**0.004**	

^*^Severe/moderate vs mild/no-DKA

### Differences in clinical presentation severity of type 1 diabetes onset

**Fig. 1:**
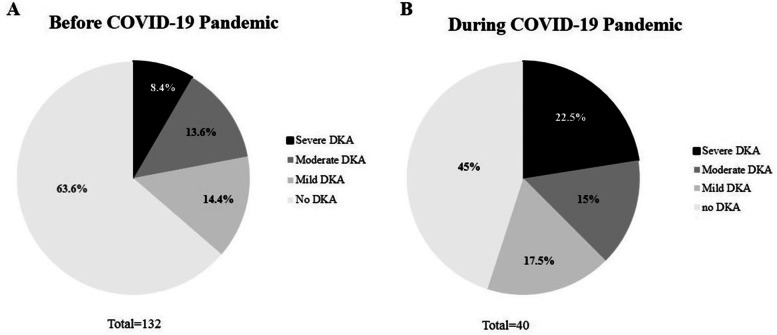
Clinical presentation of new onset Type 1 Diabetes before **A** and during COVID-19 pandemic **B**. Severe DKA: pH < 7.1; moderate DKA: pH < 7.2; mild DKA: pH < 7.3; no DKA: pH > 7.3

In Fig. 1 and Table 2 non-DKA and DKA classes of severity are reported. As shown the two groups were different in terms of proportion of cases of DKA and its severity. In details, a significant increase of DKA frequency (all DKA classes of severity) was documented in group II compared to Group I (55% vs 36.4%; *P* = 0.03). In addition, compared to Group I, Group II showed a significantly higher percentage of severe form of DKA compared to non DKA onset (11/132, 8.4% and 9/40, 22.5%, *P* = 0.01). Therefore, as expected, on the other hand the percentage of cases of type 1 diabetes onset without DKA was significant different between the two groups (*P* = 0.03), being higher in Group I (84/132, 63.6%) compared to Group II (18/40, 45%). Among those subjects with DKA onset, moderate DKA was reported in 18/132 (13.6%) and in 6/40 (15%) in Group I and Group II, respectively. As well, mild DKA was reported in 19/132 (14.4%) and in 7/40 (17.5%) in Group I and Group II, respectively. In addition, as shown in Table 2, by evaluating the two groups categorized into severe-moderate DKA and mild-non DKA classes, we found a higher proportion of subject with severe-moderate DKA in Group II (15/40, 37.5%) compared to Group I (29/132, 22%) with a significant difference between two groups (*P* = 0.04).

### Correlation analysis between pH values and the main clinical and metabolic parameters

The main data evaluating correlations between pH values and the main clinical and metabolic parameters in all children with diabetes and in the two groups divided according to the period of diabetes onset (Group I before and Group II during COVID-19 pandemic) are reported in Table 3. As shown, in the entire population and in Group I but not in Group II, pH values were significantly correlated with weight-SDS (*P* < 0.001, *P* = 0.005 and *P* = 0.13, respectively). On the other hand, BMI-SDS significantly was correlated only in the entire population (*P* = 0.01) but did not reach a significative value in the two study groups (*P* = 0.09 and *P* = 0.19, respectively). In addition, in the entire population, and in both the two groups, pH values were significantly correlated with glycemia, hemoglobin A1c, C-peptide (all *P* ≤ 0.05). Of note, in the entire population and in Group II but not in Group I, pH values were significantly correlated with Tg/HDL cholesterol ratio (*P* = 0.01, *P* = 0.002 and *P* = 0.89, respectively). In addition, in Group II but not in the entire population and in Group I pH values were significantly correlated with HDL cholesterol and tryglicerides values (*P* = 0.03 and *P* = 0.009, respectively). No correlation between pH and the other variables of interest was documented in the entire population and in the two groups, individually.

**Table 3:** Spearman correlation and multiple regression analysis between pH values and the main clinical and metabolic features in all population and in the two groups with type 1 diabetes before (Group I) and during (Group II) COVID-19 pandemic

	**Spearman Correlation**						**Multiple regression analysis**			
	**Whole population**		**Group I**		**Group II**		**Group I**		**Group II**	
	**Beta**	***P***	**Beta**	***P***	**Beta**	***P***	**Beta**	***P***	**Beta**	***P***
**Age (years)**	0.09	0.20	0.02	0.79	0.26	0.10				
**Weight-SDS**	0.28	** < 0.001**	0.24	**0.005**	0.24	0.13	0.32	**0.04**	-0.08	0.70
**Height-SDS**	0.12	0.11	0.07	0.39	0.12	0.44				
**BMI-SDS**	0.19	**0.01**	0.14	0.09	0.21	0.19	-0.29	0.06	0.09	0.61
**Glycemia (mg/dL)**	-0.28	** < 0.001**	-0.23	**0.007**	-0.47	**0.002**	-0.14	0.24	-0.14	0.43
**HbA1c (mmol/mol)**	-0.33	** < 0.001**	-0.40	** < 0.001**	-0.37	**0.01**	-0.15	0.25	-0.14	0.37
**C-peptide (ng/mL)**	0.35	** < 0.001**	0.33	**0.001**	0.31	**0.04**	0.25	0.05	0.41	**0.008**
**GAD Ab (U/ml)**	-0.01	0.90	-0.04	0.61	0.06	0.73				
**IA2 Ab (U/ml)**	-0.12	0.15	-0.15	0.11	-0.15	0.35				
**TSH (µUI/ml)**	-0.07	0.37	-0.10	0.29	-0.14	0.36				
**FT4 (ng/dL)**	0.04	0.63	-0.09	0.34	-0.10	0.53				
**Total Cholesterol (mg/dL)**	0.01	0.88	0.04	0.63	0.014	0.93				
**HDL Cholesterol (mg/dL)**	0.11	0.23	-0.002	0.96	0.36	**0.03**	0.054	0.67	0.54	**0.01**
**LDL Cholesterol (mg/dL)**	0.08	0.46	0.16	0.31	0.19	0.27				
**Tryglicerides (mg/dL)**	-0.15	0.07	-0.03	0.72	-0.43	**0.009**	-0.05	0.65	-1.84	**0.01**
**TG/HDL ratio**	-0.23	**0.01**	-0.16	0.89	-0.51	**0.002**	-0.06	0.6	1.78	**0.03**

Legend Significant values *P* ≤ 0.05. *BMI-SDS* Standard Deviation Scores-Body Mass Index; *HbA1c* Hemoglobin A1c. *IA2* Tyrosine Phosphatase antibodies IA-2. *TSH*  Thyroid-Stimulating Hormone. *fT4* free-Thyroxine.

In addition, a multiple stepwise backword regression analysis of the study population divided according to the period of type 1 diabetes onset (Group I before and Group II during COVID-19 pandemic) was performed in order to confirm the positive correlation between pH and main clinical and laboratoristic parameters. As show in Table 3 a significant correlation between pH values and c-peptide, HDL-Cholesterol, Triglycerides and Tg/HDL ratio was confirmed only for Group II. In Group I a significant correlation was documented for weight SDS while no significant association was described for the remaining variables of interest (Table 3).

## Discussion

In this study we demonstrated a significant increased risk of DKA at presentation in newly diagnosed type 1 diabetes during COVID-19 pandemic and particularly an increased prevalence of severe form of DKA compared to the previous five years. We also demonstrated in newly diagnosed subjects with type 1 diabetes during COVID-19 pandemic but not in those diagnosed during the previous five years that pH values significantly correlated positively with HDL values and indirectly with Triglycerides and TG/HDL cholesterol ratio suggesting an additional effect of lifestyle changes beside the delay in the diagnosis and these correlations were also confirmed by logistic regression analysis.

Diabetes is one of the most common chronic disease during childhood [15, 16]. Diabetic ketoacidosis is an acute and life-threatening complication of diabetes commonly associated to newly diagnosed subjects. The SEARCH for Diabetes in Youth (SEARCH) study previously reported that the DKA prevalence is one third of patients with newly onset type 1 diabetes [17]. The DKA reflects more severe pancreatic β-cell destruction and is associated with increased morbidity and mortality risk, longer hospitalization, higher insulin requirement and a worsening glycemic control over time [18]. Cherubini et colleagues showed that the worldwide prevalence of DKA at type 1 diabetes onset was increased and it is estimated around 29.9% with differences between countries; in addition, the one of the highest prevalence was highlighted in Italy (about 41%) [19, 20]. Of note, this temporal trend seems to be additionally affected by COVID-19 pandemic, as suggested by few recent studies. In particular, a Polish study found increased DKA frequency and severity during the pandemic lockdown from March 2020 until May 2020 in comparison to the same previous year [21]. On the other hand, different studies conducted in Italy during COVID-19 pandemic have described cases of delayed diagnosis of new-onset type 1 diabetes (T1D) and consequently severe DKA [22].

In a recent study conducted in Italy it is showed a different diabetes presentation as well as its severity; the study counted a fewer number of cases of type 1 diabetes onset during pandemic but a major number of children presenting with severe DKA [23]. A survey conducted in UK between March 2020 and June 2020 demonstrated an increased incidence and severity of DKA consequent to delayed medical intervention while no differences were found for the incidence of new onset type 1 diabetes [24]. In addition to these studies, by evaluating data obtained during a longer period, we demonstrated relevant differences in clinical presentation of newly cases of type 1 diabetes in children and adolescents. In fact, during COVID-19 lockdown we have documented a significant increased risk of DKA and particularly we demonstrated an increase of 19-percentage in DKA frequency among children with type 1 diabetes onset diagnosed during COVID-19 pandemic compared to the previous five years. More importantly, we also demonstrated that the severity of DKA is higher among children and adolescents diagnosed during this period. In fact, compared to those subjects diagnosed during the previous five years, newly diagnosed patients with type 1 diabetes during COVID-19 pandemic showed a significantly higher percentage of severe form of DKA (22.5% vs. 8.4%). There are fewer data about the severity of COVID-19 disease among children with type 1 diabetes [25]. It is known that DKA is strongly related to several factors and particularly to delayed diagnosis [26], which might be supposed to be certainly affected during COVID-19 pandemic. The possible causes of diagnostic delay are various; among these, there was a major attention of healthcare on COVID-19 related problems; moreover, parents avoided conducting the child to hospital and to general pediatricians for fear of acquired COVID-19 infection as demonstrated by the lower emergency room accesses during lockdown period [3]; indeed we must also considered the difficulties of moving related to the lockdown itself. These considerations strongly affect the key point of screening programmes for diabetes as well as publicity campaigns which are considered to be effective in reducing DKA prevalence at type 1 diabetes diagnosis [27, 28]. Therefore to counteract diagnostic delay, a greater awareness of type 1 diabetes [29] and health care programmes need to be supported also during those state of pandemic or long lasting emergency [30, 31]. In a recent Italian systematic review with meta-analysis was demonstrated the effectiveness of the awareness campaigns (e.g. posters, explicative letter, newspapers and magazines, mass media spots etc.) in reducing the frequency of DKA at the diagnosis of T1D in children and adolescents and are important tool for pediatricians and others to intervene in a timely manner [32].

It is known that children with type 1 diabetes are at increased risk for autoimmune diseases compared to the general population. Approximately 25% of patients are diagnosed with another autoimmune disease, being thyroid disease the most common followed by celiac disease. Compared to previous studies evaluating newly diagnosed children with type 1 diabetes [21, 23], in our study we were able to characterize several laboratory findings at diagnosis including autoimmune panel. In details, concerning thyroid autoantibodies, no significant difference was found between the two groups, although a trend toward a major percentage of subjects with autoantibodies positivity was documented in Group II. Regarding celiac disease screening, we documented a significantly higher percentage of positivity for the tTgA IgA and IgG antibodies in Group II; on the other hand, no significant difference was found for EMA antibodies. Although interesting, these findings need to be evaluated in a longitudinal setting in order to characterize any possible difference in the risk of developing other autoimmune diseases later on in life.

Previous studies have characterized lipid profile in newly diagnosed children reporting an association between impairment of lipid profile and type 1 diabetes [33]. In addition, researches have reported a correlation between TG/HDL-C and incidence of diabetes [34]. In our study, triglycerides values were significantly higher in those children with type 1 diabetes diagnosed during COVID-19 pandemic compared to Group I; similarly, a trend toward lower concentrations of HDL cholesterol levels was documented although it did not reach significant values. In addition, by performing a correlation analysis in the entire population we showed a significant association between pH and TG/HDL cholesterol ratio but not with other variables of interest. More importantly after dividing the population into the two groups according to the period of diagnosis, pH values significantly and positively correlated with HDL and negatively with triglycerides and TG/HDL cholesterol ratio in Group II but not in Group I. More importantly, these results were also confirmed by using a regression analysis thus showing a correlation between pH and lipid profile in Group II. It might be supposed that these findings might be related to the effects of lifestyle changes during COVID 19 pandemic. Relatively recent studies have shown that the Tg/HDL cholesterol ratio represents a good marker of insulin resistance in a multi-ethnic cohort of obese and overweight children being associated with both OGTT (Oral Glucose Tolerance Test) and clamp derived measures of insulin sensitivity. Therefore, further studies evaluating this marker of insulin sensitivity at diabetes onset are needed to better characterize this interesting feature. In addition, further investigations are necessary both in type 1 diabetes subjects and in all children in order to clarify the association between lifestyle habits and changes of this cardiovascular risk factor during pandemic.

The need for social isolation might increase sedentary habits and reduce physical exercise together with diet changes which are directly related to obesity and its comorbidities. In fact, during COVID 19 pandemic, recent studies have shown in children and adolescents an increased risk of obesity [35], a well-known risk factor for impaired lipid profile and increased insulin sensitivity [36]. Indeed, nutritional habits as well as physical activity may significantly influence factors related to the risk of developing DKA at diagnosis. Further and larger studies are needed to confirm our data.

One of the major limits of this study is that it is not a population-based study; consequently, the data do not reflect the general population but only a small part of it, affected by the disease. Therefore, the small sample size is due to the fact that data were obtained in a single center. The availability of new multicenter studies might further confirm our data. In addition, metabolic decompensation during DKA should be confounding factor and these data should be re-evaluated and results confirmed after achieving glycemic control. One of the major strengths of the study is the availability of data evaluating subjects with newly diagnosed type 1 diabetes during the five years before. In addition, a relevant strength is the availability of a complete laboratory assessment which added novel information in the analysis of the correlation of diabetes with other autoimmune disease.

## Conclusions

In conclusion we demonstrated that percentage of subjects with severe DKA at presentation is higher during COVID-19 pandemic which seem to be related to delayed diagnosis due to compromised access to medical care and to lifestyle habits during pandemic. Therefore, an increased efforts need to be placed by improving parental education and health system organization during this peculiar situation in order to avoid such a relevant complication of type 1 diabetes onset.

## Data Availability

The datasets used and / or analyzed during the current study are available from the corresponding author on reasonable request.

## Abbreviations

DKA: Diabetic Ketoacidosis
COVID-19: Coronavirus disease 2019
SARS-CoV 2: Severe Acute Respiratory Syndrome-Coronavirus 2
ISPAD: International Society for Pediatric and Adolescent Diabetes
BMI: Body Mass Index
SDS: Standard Deviation Score
B.E: Base Excess
HbA1c: Hemoglobin A1c
GAD65: Autoantibodies to Glutamic Acid Decarboxylase
ICA: Islet Cell Autoantibodies
IA-2: Tyrosine phosphatases IA-2 Autoantibodies
tTGA‐IgA: Tissue Transglutaminase Antibodies IgA
tTGA-IgG: Tissue Transglutaminase Antibodies IgG
EMA‐IgA: Anty-Endomysial Antibodies
TSH: Thyrotropin
fT4: Free T4
Tg-Abs: Thyroglobulin antibodies
TPO-Abs: Thyroid Peroxidase Antibodies
HDL: High-Density Lipoprotein cholesterol
LDL: Low-Density Lipoprotein cholesterol
